# Estimation of presynaptic calcium currents and endogenous calcium buffers at the frog neuromuscular junction with two different calcium fluorescent dyes

**DOI:** 10.3389/fnsyn.2014.00029

**Published:** 2015-01-07

**Authors:** Dmitry Samigullin, Nijaz Fatikhov, Eduard Khaziev, Andrey Skorinkin, Eugeny Nikolsky, Ellya Bukharaeva

**Affiliations:** ^1^Laboratory of the Biophysics of Synaptic Processes, Kazan Scientific Centre, Kazan Institute of Biochemistry and Biophysics, Russian Academy of SciencesKazan, Russia; ^2^Open Laboratory of Neuropharmacology, Kazan Federal UniversityKazan, Russia; ^3^Department of Radiophotonics and Microwave Technologies, Kazan National Research Technical University named after A. N. TupolevKazan, Russia; ^4^Department of Neurobiology and Radioelectronics, Kazan Federal UniversityKazan, Russia; ^5^Department of Medical and Biological Physics, Kazan State Medical UniversityKazan, Russia

**Keywords:** neuromuscular junction, presynaptic calcium transient, inward calcium current, endogenous calcium buffers, mathematical modeling

## Abstract

At the frog neuromuscular junction, under physiological conditions, the direct measurement of calcium currents and of the concentration of intracellular calcium buffers—which determine the kinetics of calcium concentration and neurotransmitter release from the nerve terminal—has hitherto been technically impossible. With the aim of quantifying both Ca^2+^ currents and the intracellular calcium buffers, we measured fluorescence signals from nerve terminals loaded with the low-affinity calcium dye Magnesium Green or the high-affinity dye Oregon Green BAPTA-1, simultaneously with microelectrode recordings of nerve-action potentials and end-plate currents. The action-potential-induced fluorescence signals in the nerve terminals developed much more slowly than the postsynaptic response. To clarify the reasons for this observation and to define a spatiotemporal profile of intracellular calcium and of the concentration of mobile and fixed calcium buffers, mathematical modeling was employed. The best approximations of the experimental calcium transients for both calcium dyes were obtained when the calcium current had an amplitude of 1.6 ± 0.08 pA and a half-decay time of 1.2 ± 0.06 ms, and when the concentrations of mobile and fixed calcium buffers were 250 ± 13 μM and 8 ± 0.4 mM, respectively. High concentrations of endogenous buffers define the time course of calcium transients after an action potential in the axoplasm, and may modify synaptic plasticity.

## Introduction

Calcium ions (Ca^2+^) play a leading role in the initiation, maintenance, and plasticity of neurotransmitter release at chemical synapses in both the central and peripheral nervous systems (Llinás et al., 1992; Chow et al., 1994; Augustine, 2001; Burnashev and Rozov, 2005; Schneggenburger and Neher, 2005; Pang and Südhof, 2010). The influx of Ca^2+^ into the nerve terminal following the action potential initiates the release of neurotransmitter and the subsequent postsynaptic potential. The properties of this potential reflect the magnitude of neurotransmitter release and the sensitivity of the postsynaptic membrane to the neurotransmitter.

In some synapses, the Ca^2+^ current can be measured directly (Borst and Sakmann, 1996, 1998; Yazejian et al., 1997), but such recordings from the frog neuromuscular junction are problematic because the small diameter (1–2 μm) and substantial length (100–500 μm) of the nerve terminal prohibits the use of the patch clamp and two-electrode voltage clamp techniques. Recordings with a loose macropatch clamp formed under the nerve perineurial sheath (Anderson et al., 1988), or with a macropatch electrode attached to the membrane as close as possible to a release site (Brigant and Mallart, 1982), have been accomplished only under non-physiological conditions, such as in the presence of K^+^-channel blockers.

Alternatively, optical methods may be employed to estimate the action-potential-induced Ca^2+^ influx and the subsequent distribution of Ca^2+^ ions in the axoplasm. The basis of these methods is the recordings of fluorescence transients which arise as a result of changes in the fluorescence intensity of specific calcium-sensitive dyes that interact with Ca^2+^ ions (DiGregorio and Vergara, 1997; Sabatini and Regehr, 1998; Suzuki et al., 2000; Sabatini et al., 2002; Luo et al., 2011).

The profile of intracellular Ca^2+^ distribution, and consequently the parameters of the fluorescence signal, is defined by the calcium influx and by the concentration of endogenous calcium-binding proteins (Ahmed and Connor, 1988; Burnashev and Rozov, 2005; Collin et al., 2005; Lin et al., 2005; Kreiner and Lee, 2006; Muller et al., 2007). After entering through voltage-gated channels, 95% of the Ca^2+^ ions bind to binding sites located within 10–50 nm of the site of entry (Neher, 1995). Mobile calcium buffers are represented by soluble Ca^2+^-binding proteins of the EF-hand family, including parvalbumins, calbindin-D9k, calbindin-D28k, calretinin, calmodulin, and proteins of the S100 family (Schwaller, 2010), or else by small molecules such as ATP and GTP which affect the diffusion of Ca^2+^ and define their operational distance (Feher et al., 1989; Zhou and Neher, 1993). Fixed endogenous Ca^2+^ buffers are associated with cellular organelles or elements of the cytoskeleton (Gabso et al., 1997; Muller et al., 2005). Fixed buffers slow the diffusion of Ca^2+^ and prolong the lifetime of high Ca^2+^ levels near the release zone (Zhou and Neher, 1993; Kits et al., 1999; Matveev et al., 2004). But for many peripheral synapses, including the frog neuromuscular junction, many of the details of the regulation of axoplasmic Ca^2+^ and of the properties of endogenous calcium buffers in terminals remain unknown.

In previous work, we have compared experimental and model-based approaches to show that the presence of endogenous fixed calcium buffers in the motor nerve terminal of frogs can explain the desynchronization of neurotransmitter release under conditions of low extracellular calcium and synchronization of quantal release in the presence of a mobile buffer (Samigullin et al., 2005; Bukharaeva et al., 2007; Gilmanov et al., 2008). In the present work, we measure fluorescence transients activated by a single nerve impulse in the motor nerve terminal of a frog using two calcium-sensitive fluorescent dyes with different affinities to calcium. We compare these fluorescence transients with the simultaneous measurement of the postsynaptic response of a muscular fiber. We then use mathematical modeling to estimate the parameters of the calcium current and the contribution of the endogenous calcium buffers of different mobilities to the spatial distribution of Ca^2+^ in the axoplasm.

## Materials and methods

### Preparation and solutions

The animals (frogs, *Rana ridibunda*) were anesthetized with ether prior to being decapitated in accordance with the European Communities Council Directive (November 24, 1986; 86/609/EEC). The protocol of the experiments was approved by the Animal Care and Use Committee of Kazan State Medical University. An isolated neuromuscular preparation of the *musculus cutaneous pectoris* was continuously perfused with Ringer solution (in mM): NaCl 113.0, KCl 2.5, CaCl_2_ 1.8, and NaHCO_3_ 3.0 (pH 7.2–7.4). The temperature was set at 20.0 ± 0.3°C. D-tubocurarine (5 μM) was added to the solution to prevent muscle contractions in response to nerve stimulation.

### Calcium dye loading

Two fluorescent calcium dyes of different affinities were used: the low-affinity Magnesium Green (MG), which has dissociation constant *K*_d_ = 19 μM, and the high-affinity Oregon Green BAPTA-1 (OGB-1), whose dissociation constant is *K*_d_ = 0.17 μM (Maravall et al., 2000). The loading concentration of the fluorescent dyes was 50 mM. All dyes were obtained from Molecular Probes (Eugene, OR, USA).

The loading of the dyes was carried out through the nerve stump, as described by Peng and Zucker (1993) and Wu and Betz (1996). After this procedure, all terminals in the proximal part of the nerve trunk had sufficient basal levels of fluorescence. It has been established that the approximate intra-terminal concentration of dyes loaded into a nerve by this method is 40–150 μ M (Suzuki et al., 2000). The loading procedure does not have any appreciable influence on the amplitude of the postsynaptic response or on the frequency of the miniature end-plate potentials (Wu and Betz, 1996).

### Recording of the fluorescent signals of the single action potentials

Fluorescence signals were monitored using a photometric system based on an Olympus BX-51 microscope with water immersion objective (40×, NA = 0.8) and a high-sensitivity photodiode (S1087, Hamamatsu, Japan) (Samigullin et al., 2010). To choose the area for recording the fluorescence, we used the optical viewfinder (Till Photonics, Munich, Germany). Light excitation at a wavelength of 488 nm was generated by Polychrome V (Till Photonics, Munich, Germany). To minimize bleaching of the dye and to the lower background fluorescence, the recording area of the nerve terminal was restricted using an iris diaphragm. Illumination was gated by a shutter with a typical exposure time of 400 μs and a delivery rate of 0.5 Hz. The signals from the photodiode were digitized with a Digidata 1200B analog-to-digital converter (Axon Instruments, California, USA). The recording of fluorescence and the synchronization of the illumination and stimulation was controlled by WinWCP software (Strathclyde University, Glasgow, UK). The change in fluorescence was represented as ΔF/F_0_ (the change in fluorescence intensity relative to the background fluorescence as a percentage). For each experiment, about 60 fluorescent responses were recorded and averaged.

The time parameters of the fluorescence signals depend on the interaction of the dye with the calcium—specifically, on the speed of the forward and reverse reactions of the calcium–dye complex formation. The speeds of the forward reaction of the low-affinity (MG) and high-affinity (OGB-1) dyes differ (see Table 1). The dissociation rate of the calcium–dye complex is almost 30 times lower for OGB-1 than for MG, which explains the longer rise time and decay time observed with OGB-1.

**Table 1:** **Parameters of the model**.

**Parameter**	**Value**	**References**
Intracellular concentration of calcium [Ca]_in_	0.05 μM	Helmchen et al., 1996
Calcium diffusion (*D*_*Ca*_)	220 μm^2^ s^−1^	Albritton et al., 1992
Endogenous fixed buffer (*k*_on_)	100 μM^−1^ s^−1^	Xu et al., 1997
Endogenous fixed buffer (*k*_off_)	10^4^ s^−1^	Xu et al., 1997
Endogenous fixed buffer concentration	2000–12000 μM	^**^
Endogenous mobile buffer (*k*_on_)	6 μM^−1^ s^−1^	Schwaller, 2010
Endogenous mobile buffer (*k*_off_)	1 s^−1^	Schwaller, 2010
Endogenous mobile buffer diffusion (*D*_Bmob_)	43 μm^2^ s^−1^	Schmidt et al., 2003
Endogenous mobile buffer concentration	50–500 μM	^**^
Magnesium Green (*k*_on_)	90 μM^−1^ s^−1^	Zhao et al., 1996
Magnesium Green diffusion	15 μm^2^ s^−1^	Zhao et al., 1996
Magnesium Green dissociation constant	19 μM	Zhao et al., 1996
Magnesium Green concentration	100 μM	Suzuki et al., 2000
Oregon Green BAPTA-1 *k*_on_	400 μM^−1^ s^−1^	Saftenku, 2009
Oregon Green BAPTA dissociation constant	0.17 μM	Maravall et al., 2000
Oregon Green BAPTA diffusion	15 μm^2^ s^−1^	Saftenku, 2009
Oregon Green BAPTA concentration	100 μM	Suzuki et al., 2000
Maximum pump velocity (*V*_*max*_)	3.8^.^10^−4^ μM. cm^−2^ s^−1^	^**^
Pump dissociation constant (*K*_*pm*_)	0.83 μM	Sala and Hernandez-Cruz, 1990
Leak rate (*K*_*leak*_)	1.2 10^−10^ cm^−2 s^−1^^	^**^
Extracellular concentration of calcium [Ca]_*extra*_	1800 μM	Experimental conditions
Width of calcium current (σ)	0.0005 s	^**^
Amplitude of calcium current (*i*_*Ca*_)	1.6 pA	^**^

** Obtained from modeling parameters.

### Electrophysiological recording of nerve-action potentials and end-plate currents

Suprathreshold stimuli of 0.2 ms duration were applied to the nerve at 0.5 Hz through a suction electrode. The nerve-action potentials and extracellular end-plate currents were recorded using heat-polished Ringer-filled extracellular pipettes with tip diameters of 20 μm and resistances of 1–3 MΩ. For optimal contact with the preparation, the pipette tip was beveled at 35° (Vyshedskiy and Lin, 2000). The calcium transients were recorded in the same region where the extracellular pipette was placed. The electrical and optical signals were sampled simultaneously at 3-μs intervals by a 12-bit analog–digital converter (Digidata 1200B, Axon Instruments, California, USA), stored on a computer, and processed in AxoScope (Axon Instruments, California, USA).

### Computer modeling of intracellular calcium concentrations

Experimental and theoretical studies have shown that, in the frog nerve terminal, a presynaptic action potential only opens a few calcium channels, and that the interaction of calcium influxes through different channels is low (Wachman et al., 2004; Luo et al., 2011). Experiments in non-mammalian synapses indicate that calcium channels are tightly coupled to the sites where synaptic vesicles are docked; the opening of a single Ca^2+^ channel (producing a Ca^2+^ nanodomain; Yoshikami et al., 1989; Augustine, 1990; Stanley, 1991) is sufficient to initiate vesicular fusion. We therefore neglected non-linear interactions of currents through the channels and considered only one channel in the model of one active zone, with the assumption that the total Ca^2+^ current is the linear summation of currents through the individual channels. Such a presentation of the Ca^2+^ channel as a point source on the presynaptic membrane is common for modeling exocytotic release (Smith, 1996; Bauer, 2001; Gilmanov et al., 2008).

The terminal was modeled as a cylinder with height of 2 μm and a diameter of 2 μm, with the calcium channel as a point source in its center. The height of frog's neuromuscular terminal (the height of the cylinder in the model—Figure S1 in the Supplementary Materials) was taken from Birks et al. (1960) and Pawson et al. (1998). In addition to fluorescent dye molecules in the cytoplasm, the presence of mobile and fixed buffers was also assumed (Klingauf and Neher, 1997). The interaction of Ca^2+^ in the terminal with the fluorescent dye and endogenous Ca^2+^ buffers can be described by the equation:

(1) $$Ca\text{ + }B_{n}\begin{array}{c} k_{B_{n}}^{\text{on}}\\ ⇄\\ k_{B_{n}}^{\text{of}} \end{array}CaB_{n},$$

where *k*^on^_*B*_*n*__ and *k*^of^_*B*_*n*__ are the association and dissociation rate constants and B_*n*_ (where *n* = *d*, *fix*, *mob*, referring to the fluorescent dye, fixed endogenous buffer, and mobile endogenous buffer, respectively). Assuming mass action kinetics and Fickian diffusion, we established a system of seven partial differential equations (PDE):

(2) $$\begin{array}{l} \frac{\partial [\text{Ca}]}{\partial t}\;=\;D_{Ca}\;\cdot △\left[Ca\right]+i_{Ca}\;\cdot \delta \left(x\;-\;x_{0},y\;-\;y_{0},z\;-\;z_{0}\right)\\ \;-k_{B_{fix}}^{\text{on}}\;\cdot \;\left[Ca\right]\;\cdot \;\left[B_{fix}\right]+k_{B_{fix}}^{\text{of}}\;\cdot \;\left[CaB_{fix}\right]-k_{B_{mob}}^{\text{on}}\;\cdot \;\left[Ca\right]\cdot \\ \;\left[B_{mob}\right]\;+\;k_{B_{mob}}^{\text{of}}\;\cdot \;\left[CaB_{mob}\right]-k_{d}^{\text{on}}\;\cdot \;\left[Ca\right]\;\cdot \;\left[B_{d}\right]\\ \;+k_{d}^{\text{of}}\;\cdot \;\left[CaB_{d}\right],\\ \frac{\partial [B_{fix}]}{\partial t}\;=\;-k_{B_{fix}}^{\text{on}}\;\cdot \;\left[Ca\right]\;\cdot \;\left[B_{fix}\right]+k_{B_{fix}}^{\text{of}}\;\cdot \;\left[CaB_{fix}\right],\\ \frac{\partial [CaB_{fix}]}{\partial t}\;=\;k_{B_{fix}}^{\text{on}}\;\cdot \;\left[Ca\right]\;\cdot \;\left[B_{fix}\right]-k_{B_{fix}}^{\text{of}}\;\cdot \;\left[CaB_{fix}\right],\\ \frac{\partial [B_{mob}]}{\partial t}\;=\;D_{B_{mob}}\;\cdot △\left[B_{mob}\right]-k_{B_{mob}}^{\text{on}}\;\cdot \;\left[Ca\right]\;\cdot \;\left[B_{mob}\right]\\ \;+k_{B_{mob}}^{\text{of}}\;\cdot \;\left[CaB_{mob}\right],\\ \frac{\partial [CaB_{mob}]}{\partial t}\;=\;D_{B_{mob}}\;\cdot △\left[CaB_{mob}\right]+k_{B_{mob}}^{\text{on}}\;\cdot \;\left[Ca\right]\;\cdot \;\left[B_{mob}\right]\\ \;-k_{B_{mob}}^{\text{of}}\;\cdot \;\left[CaB_{mob}\right],\\ \frac{\partial [B_{d}]}{\partial t}\;=\;D_{d}\;\cdot \;△\left[B_{d}\right]-k_{d}^{on}\;\cdot \;\left[Ca\right]\;\cdot \;\left[B_{d}\right]\;+\;k_{d}^{of}\;\cdot \;\left[CaB_{d}\right],\\ \frac{\partial [CaB_{d}]}{\partial t}\;=\;D_{d}\;\cdot \;△\left[CaB_{d}\right]+k_{d}^{\text{on}}\;\cdot \;\left[Ca\right]\;\cdot \;\left[B_{d}\right]-k_{d}^{\text{of}}\;\cdot \;\left[CaB_{d}\right], \end{array}$$

where *D*_*Ca*_, *D*_*Bmob*_, and *D*_*d*_ are the diffusion constants for free Ca^2+^, the mobile buffer, and the fluorescent dye, respectively, δ (*x* − *x*_0_, *y* − *y*_0_, *z* − *z*_0_) is the Dirac delta function denoting the calcium channel, where *x*_0_, *y*_0_, *z*_0_ are coordinates of the center of cylinder base. Three-dimensional geometry with Cartesian coordinates was used in our modeling.

The action-potential-induced calcium influx *i*_*Ca*_ was modeled as a Gaussian-shaped calcium current, as measured in the presynaptic terminals (Llinás et al., 1976):

(3) $$i_{Ca}=A\;\cdot \;e^{-\frac{{\left(t-t_{peak}\right)}^{2}}{\text{2}\cdot \sigma^{2}}},$$

where *A* is current amplitude and *t_peak_* is the time of the peak of the Gaussian calcium current. In this model, we have used the maximum of the presynaptic action potential as the starting point of the calcium current, since it is precisely the depolarization caused by the action potential that opens the presynaptic calcium channels. The parameter σ defines the width of the calcium current, for which we have also calculated the parameter *t*_1/2_ (the width of the current at half-amplitude). The parameters *t*_1/2_ and σ are connected by the equation:

(4) $$t_{\frac{1}{2}}=2\;\cdot \;\sqrt{\text{2 }\cdot \;\ln\left(\text{2}\right)\;}\cdot \;\sigma$$

We have taken into account two additional calcium currents that pass through the cylinder base: the current representing the work of the membrane pumps and a leak current:

(5) $$J=-V_{\text{max}}\;\cdot \;\frac{\left[Ca\right]}{\left[Ca\right]\text{+}K_{pm}}\text{+}K_{\text{leak}}\;\cdot \;\left(\left[Ca_{\text{extra}}\right]\text{–}\left[Ca\right]\right),$$

where *V*_*max*_ is the maximum pump velocity, *K_*pm*_* is the pump dissociation constant, *K*_*leak*_ is the membrane conductivity for calcium, and [*Ca*_*extra*_] is the extracellular concentration of calcium.

For all species, the flux through all boundaries was null, i.e.,

n · (−D∇[A])=0,

where ***n*** is normal to the surface cylinder, *A* is the designation of some species in the model. However, the current *J* through the cylinder base containing the calcium channel was present in the model throughout all the simulation. So for this base only:

−n · (−D∇[Ca])=J.

The initial conditions for all species were the equilibrium concentrations listed in Table 1.

The system of PDEs was solved using the finite element method, taking 0.1 μm as the size of elements in the volume of a cylinder and 0.1 nm near the point source, using Comsol Multiphysics 3.5 and Matlab.

The model parameters are specified in Table 1. To model the Ca^2+^ transients, we varied the concentrations of the mobile and fixed buffers, as well as the amplitude and duration of the inward Ca^2+^ current. We employed a human-based approach here, but the variations of all free parameters were made in the full physiological range in increments of 5% of the range. We then compared the resulting fluorescence signal of the model with the signal observed in experiments. The theoretical fluorescence signal was used to derive the total number of calcium–dye complexes in the model at each moment in time, as the fluorescence of these molecules gives rise to the fluorescence signal registered in the experiment. The concentration range of the mobile buffer was 50–500 μM, while the concentration range of the fixed buffer was 2000–12000 μ M. These parameters correspond to the values established for the nerve terminals in synapses of a crayfish (Vyshedskiy and Lin, 2000) and in synapses of the central nervous system (Wu and Saggau, 1994). The selection of parameters that provides the best agreement of the modeling and experimental curves with the calcium transient for the differing concentrations of fixed and mobile buffers are shown on Figure S2 in the Supplementary Materials.

## Results

### Action potential induced responses of Ca^2+^ dyes with differing affinities for Ca^2+^

**Figure 1:**
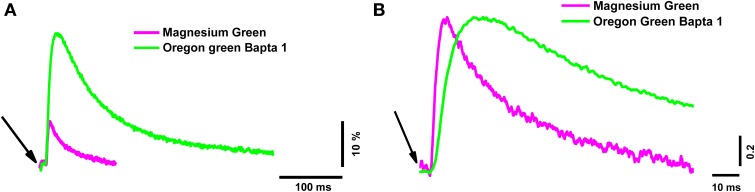
**The time course of calcium transients obtained in the nerve terminal from one electrical stimulus with two different dyes**. Magnesium Green (magenta line) and Oregon Green BAPTA-1 (green line). **(A)** Averaged unnormalized calcium transients represented as ΔF/F_0_. **(B)** Averaged calcium transients normalized to the maximum value to compare the time parameters. The averages were calculated from 60 responses. The beginning of the short (200 μs) electrical stimulus of the nerve terminal is indicated with arrows.

The peak of the fluorescence signal in nerve terminals loaded with the low-affinity dye MG was about 2.6 times smaller than in terminals loaded with the high-affinity dye OGB-1 (see Table 2 for details). The kinetics of the response of the low-affinity dye was significantly faster than the response of the high-affinity dye (Table 2 and Figure 1). Similar differences have been observed in synapses of the central nervous system (Regehr and Atluri, 1995) and in developing neuromuscular junctions of *Xenopus* (DiGregorio and Vergara, 1997). As described in the Materials and Methods section, the time parameters of fluorescence signals depend on the speed of the forward and reverse reactions of the formation of the calcium–dye complex. Thus, identical inward calcium currents evoked in nerve terminals loaded with dyes with different properties are accompanied by fluorescence signals with different amplitude and temporal properties. It is therefore impossible to accurately determine the behavior of the intracellular free calcium based on the response of only one fluorescent dye.

**Table 2:** **Summary of the characteristics of OGB-1 and MG fluorescence transients**.

	**Peak ΔF/F_**0**_ (%)**	**Rise time 20–80% (ms)**	**τ, (ms)**
OGB-1	26 ± 6 (*n* = 9)	6.00 ± 0.36 (*n* = 9)	89.07 ± 7.34 (*n* = 9)
Magnesium Green	10 ± 3 (*n* = 6)	2.08 ± 0.23 (*n* = 6)	34.15 ± 4.06 (*n* = 6)

Data are presented as Mean ± SE, and n is the number of measured nerve–muscle junctions. Peak ΔF/F_0_ is the averaged maximum ΔF/F_0_ attained at a single spot location.

### Simultaneous recording of presynaptic fluorescence signal and postsynaptic electrical response

**Figure 2:**
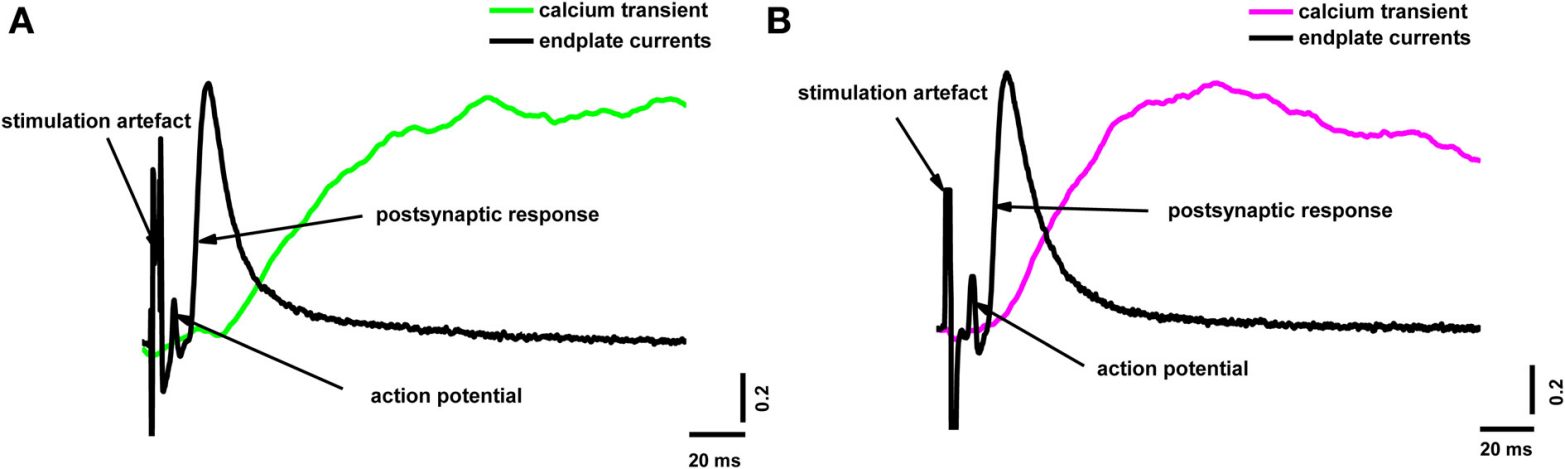
**Simultaneous recording of end-plate currents and fluorescence calcium transients caused by a single action potential in a terminal loaded with two different dyes**. **(A)** Fluorescence calcium transient for Oregon Green BAPTA-1(green line), end-plate current (black line); **(B)** fluorescence calcium transient for Magnesium Green (magenta line), end-plate current (black line). The figure shows typical experimental recordings for each dye obtained from the average of 60 responses per experiment. Traces are normalized to the maximum values. End-plate currents were inverted to allow comparison with fluorescence signals.

To reveal the correlation between the amplitude of the presynaptic calcium influx and neurotransmitter release, we simultaneously recorded the presynaptic fluorescence signal and the end-plate potential. Figure 2 shows the calcium signals and the extracellulary recorded action potentials of the nerve terminal and end-plate currents. It is clearly visible in Figure 2 that the postsynaptic current developed much faster than the fluorescence signal; in essence, the postsynaptic current ended before the fluorescence signal even reached its maximum. This means that, although the fluorescence signal occurs as a consequence of the interactions of calcium ions with the indicator in the nerve terminal of the frog neuromuscular junction, it does not directly reflect the change in Ca^2+^ concentration in the active release zones.

Because electrophysiological techniques cannot be used to directly record calcium currents in the frog terminal, we used the mathematical model described in the Materials and Methods section to deduce the parameters of the inward calcium current that reproduces the observed fluorescence transient, and also in order to define a profile of calcium concentration changes at different points of the terminal, including the near-membrane space.

### Model parameters and quantification of the endogenous calcium buffer concentration

**Figure 3:**
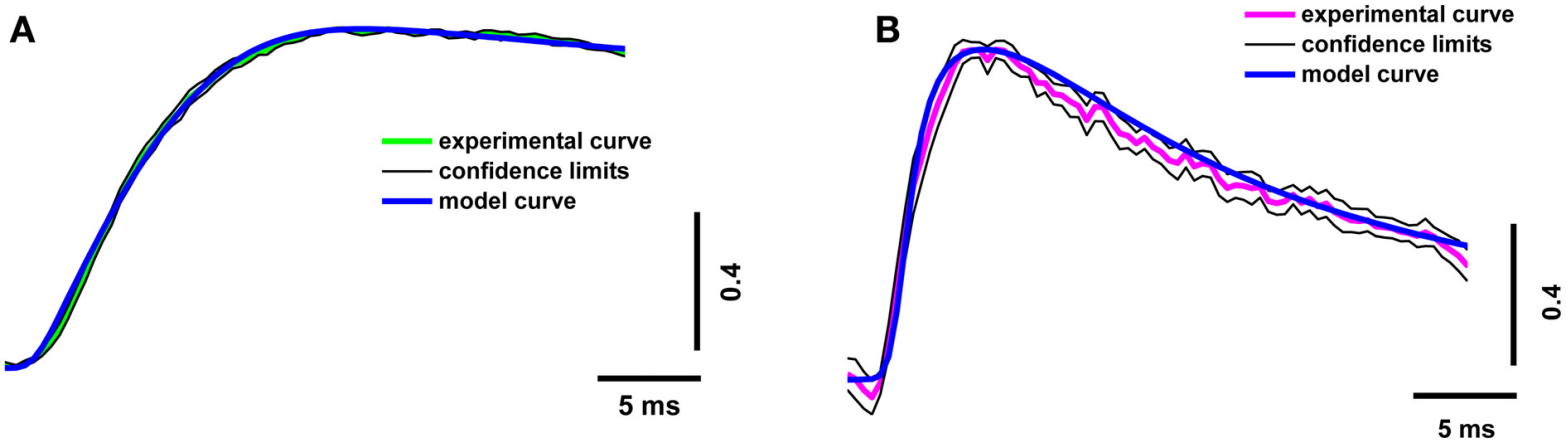
**Superposition of experimental and model calcium transients in terminals loaded with two different dyes**. **(A)** Experimental curve for Oregon Green BAPTA-1(green line), model calcium transient (blue line), confidence interval for experimental curve (thin black line); **(B)** experimental curve for Magnesium Green (magenta line), model calcium transient (blue line), and confidence interval for experimental curve (thin black line). Traces are normalized to the maximum value.

In order to increase the reliability of the model, we compared the theoretical and experimental fluorescence transients obtained with the different dyes and different regimes of nerve stimulation. As specified in Table 1, which shows the model parameters, the amplitude and duration of the calcium current and the concentrations of the fixed and mobile buffers were the variable parameters in the model. These were selected so that the model best reproduced the kinetics of the fluorescence transient for both dyes. We found that a presynaptic calcium current with amplitude 1.60 ± 0.08 pA and half-width 1.20 ± 0.06 ms resulted in the best agreement between the model and the experimental fluorescence transients. The presence in the axoplasm of a mobile calcium buffer at a concentration of 250 ± 13 μM and of fixed buffer at 8.0 ± 0.4 mM could explain the observed temporal differences in the measured fluorescence signal, compared with the postsynaptic response. We have checked additionally that, when all four model-derived parameters are within the intervals shown in Table 1, the shape of simulated fluorescence reproduces the shape of the experimental fluorescence (taking into account the dispersion of the experimental data; Figure 3). Figure S2 in the Supplementary Materials demonstrate the selection of parameters that provides the best agreement of the modeling and experimental curves with the calcium transient for the differing concentrations of fixed and mobile buffers.

### Fluorescence transients during rhythmic stimulation of the motor nerve

The neuromuscular junction *in vivo* operates in a mode of rhythmic activity, so it is important to estimate the intracellular calcium kinetics not only in response to individual stimuli, but also during repetitive stimulation. We estimated the properties of the calcium transients for the three signals occurring in response to a 50-Hz train of action potentials in the presence of dyes of both types.

**Figure 4:**
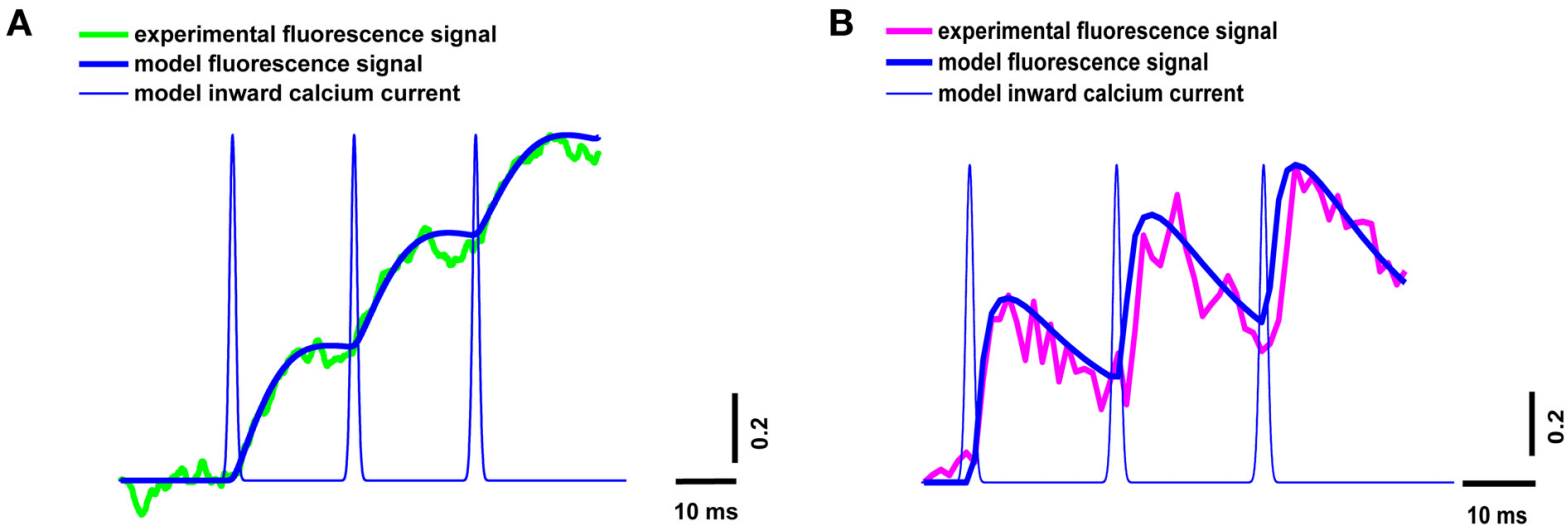
**Superposition of experimental and model calcium transients in the terminal loaded with two different dyes during a stimulus train consisting of three signals at 50 Hz**. **(A)** Experimental curve for Oregon Green BAPTA-1(green line), model calcium transient (thick blue line), model inward calcium current (thin blue line); **(B)** experimental curve for Magnesium Green (magenta line), model calcium transient (thick blue line), model inward calcium current (thin blue line). Traces are normalized to the maximum value.

The parameters of the simulated fluorescence response were in a good agreement with those of the experimental response obtained using the high- and low-affinity dyes (Figure 4), thus confirming the validity of the model.

### The estimation of calcium concentration in the near-membrane space

**Figure 5:**
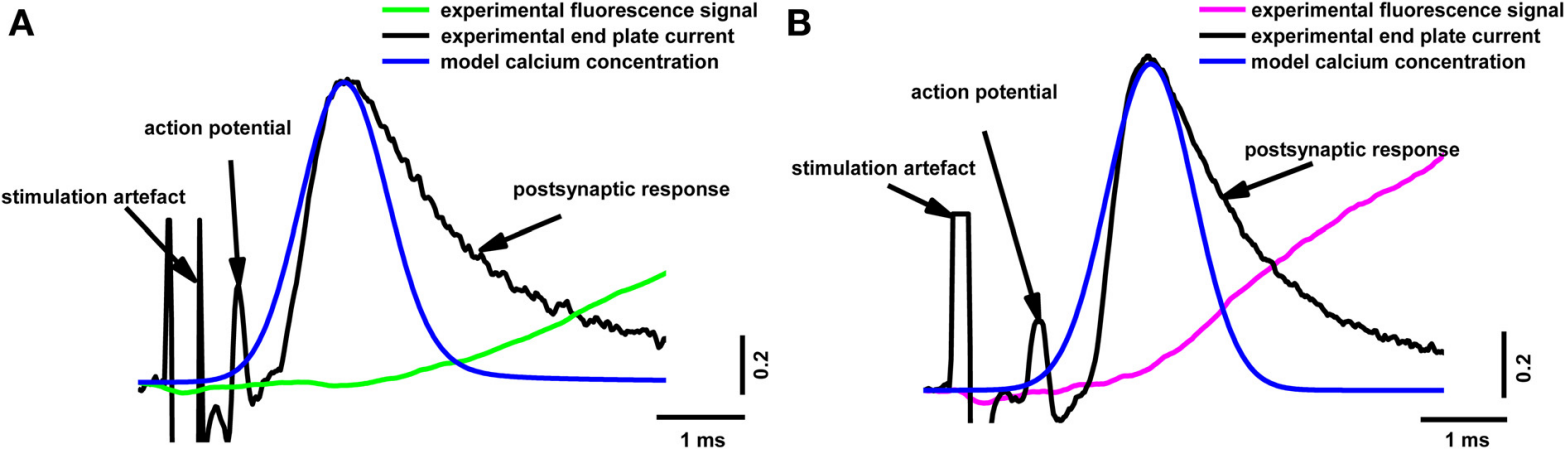
**Superposition of the calculated calcium concentration dynamics at a distance of 15 nm from the calcium channel, with simultaneous recording of end-plate current and calcium transients induced by a single action potential in terminals loaded with two different dyes**. **(A)** Fluorescence calcium transient for Oregon Green BAPTA-1(green line), experimental end-plate current (black line), model calcium concentration (blue line); **(B)** fluorescence calcium transient for Magnesium Green (magenta line), experimental end-plate current (black line), model calcium concentration (blue line). Traces are normalized to the maximum value. Experimental data are the same as in Figure 2.

Our model allowed us to calculate the calcium distribution in different parts of the terminal. It is particularly important to estimate Ca^2+^ concentration in the near-membrane region, since this determines the number of released quanta and the kinetics of neurotransmitter release. Figure 5 shows the calculated dynamics of the calcium concentration at a distance of 15 nm from the calcium channel, in comparison with the experimental postsynaptic current and fluorescent signal. The beginning of the rise in the calcium concentration in the near-membrane space preceded the postsynaptic response, indicating that the real time between the beginning of the calcium response in the active zone and the beginning of the postsynaptic current (about 600 μs) was sufficient for the activation of the exocytosis machinery, the release of neurotransmitter, the activation of the postsynaptic receptors, and the opening of the postsynaptic ion channels.

## Discussion

In a nerve terminal loaded with calcium-sensitive dye, calcium ions entering through Ca^2+^ channels bind to the dye, producing a change in its fluorescence intensity. Analysis of Ca^2+^-related fluorescent signals reveals the dynamics of calcium concentration in the nerve terminal which demonstrate, for example, that synaptic facilitation caused by the accumulation of intracellular calcium outlasts the increase in Ca^2+^ in the terminal (Regehr and Atluri, 1995). Analysis of Ca^2+^-dependent fluorescence changes has also been used to identify the types of voltage-gated calcium channels that participate in neurotransmitter release, and to clarify the degree of cooperativity of Ca^2+^ channels (Wu and Saggau, 1994; Mintz et al., 1995; Tsentsevitsky et al., 2014). While electrophysiological recording of Ca^2+^ currents provides a more direct measure (Vyshedskiy and Lin, 2000), the morphological features of some synapses—such as the neuromuscular junctions of mice, frogs, and other vertebrates—render the use of voltage clamp methods difficult. Therefore, the use of calcium-sensitive dyes is an alternative for estimating calcium influx into the nerve terminal.

The fluorescent signal had a very slow rise compared with the postsynaptic response, and presumably the calcium current. Similar observations have been made in the neuromuscular synapses of lizards and frogs (David et al., 1997; DiGregorio and Vergara, 1997; Bélair et al., 2005). However, this temporal difference between the beginning of the fluorescence signal and the postsynaptic potential has not always been observed, and it has generally been assumed that the fluorescence transient reflects the change in calcium concentration in the terminal, while its first derivative reflects the calcium current (Sabatini and Regehr, 1998). The lag observed in our study could be interpreted, for example, as a consequence of the presence of a high concentration of fixed buffer, resulting in slow diffusion of calcium into the terminal (Lin et al., 2005). In this case, calcium entering the terminal interacts directly with proteins of the exocytosis machinery to trigger neurotransmitter release, and only subsequently does the slow diffusion of calcium cause the formation of calcium–dye complexes and an appreciable fluorescence signal. Because we recorded the fluorescence from the entire volume of the terminal, the slow kinetics of the signal are likely to reflect a process of slow calcium distribution. Another reason why the postsynaptic current in Figure 2 develops earlier than the fluorescence signal is that the frog neuromuscular junction is a fast synapse (as opposed to slow NMDA, for example) with relatively short synaptic delays and quickly rising and falling postsynaptic currents. For the frog neuromuscular junction, the time between neurotransmitter release and the start of the postsynaptic current is very short (about 300–500 μs; Bukharaeva et al., 2002). In some other synapses, the slow activation of postsynaptic channels can mask the paradox shown in Figure 2. The current through these channels grows at a similar speed to the fluorescence signal (for example, Vyshedskiy and Lin, 2000) due to the slow activation.

Our mathematical model permits us to clarify the observation that the postsynaptic current develops much faster than the fluorescence signal. After the opening of the voltage-gated channels, Ca^2+^ enters the terminal and, in the process of diffusing away from the entry site, binds to the dye molecules that are distributed throughout the volume of the terminal. The rising phase of the fluorescent signal of the calcium–dye complex in the area recorded by the photodiode depends on the speed of calcium diffusion, which in turn depends on the concentration and properties of endogenous buffers. Endogenous calcium buffers play the leading role in the spatiotemporal distribution of Ca^2+^ (Sala and Hernandez-Cruz, 1990; Nowycky and Pinter, 1993). After the action-potential-triggered Ca^2+^ influx through the voltage-dependent channels, Ca^2+^ ions bind to the buffers within 10–50 nm of the entry site (Augustine and Neher, 1992; Neher, 1995). Further diffusion of Ca^2+^ depends mainly on the degree of mobility of Ca^2+^-binding sites. Immobile buffers, which are localized on cellular organelles or cytoskeleton elements, have a slower speed of diffusion than free calcium. Such fixed buffers delay diffusion, because they take up and bind calcium ions for some time, thus reducing the speed of calcium distribution within the space of the terminal (Zhou and Neher, 1993; Kits et al., 1999). This slowing of diffusion has important consequences for any intracellular calcium-dependent process.

**Figure 6:**
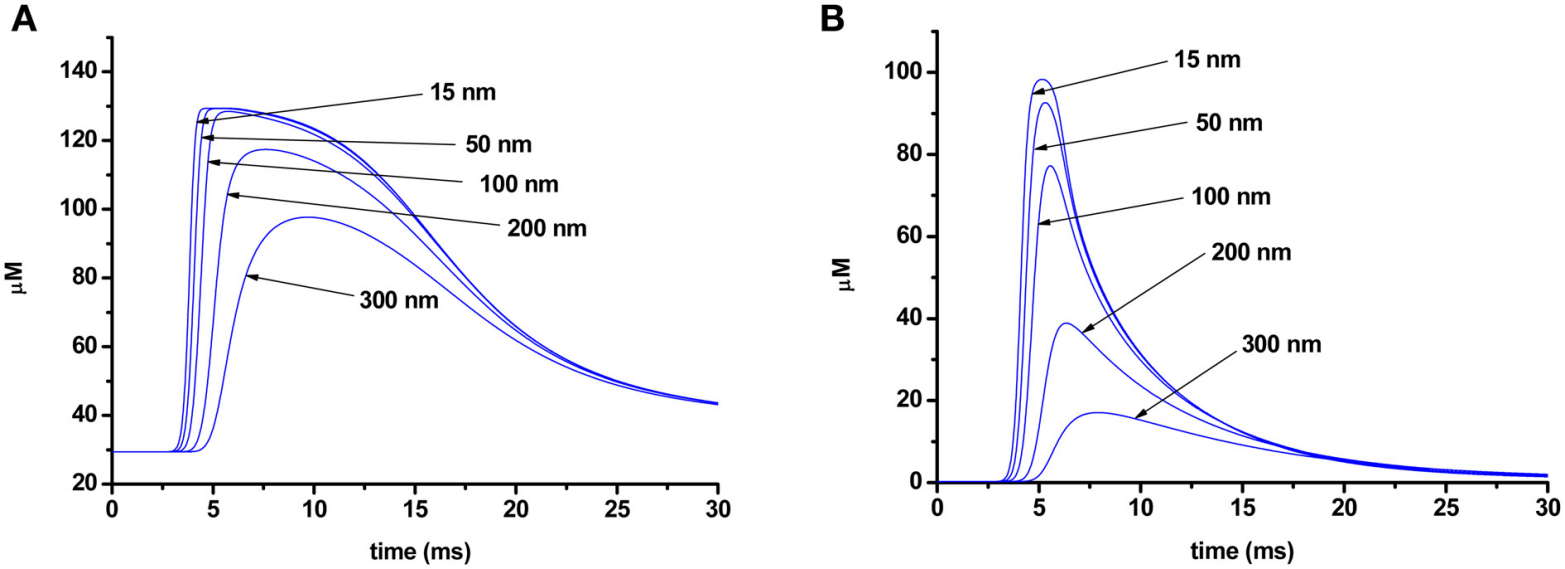
**Change in concentration of a calcium–dye complex at different distances from the point of calcium entry (15, 50, 100, 200, 300 nm) in the terminal loaded with Oregon Green BAPTA-1 (A) or Magnesium Green (B)**.

As Ca^2+^ slowly diffuses through the terminal, dissociation of the calcium–dye complexes begins, and the duration of the rising phase of the fluorescent signal also depends on the speed of this process. Figure 6 shows the changes in the concentration of a calcium–dye complex at various points of a terminal for the two different dyes. Dissociation of the calcium–dye complex is expected to occur faster in the case of a low-affinity dye, as the rate of the reverse reaction is greater than for the high-affinity dye. The phase of the signal rise ends when the rate of formation of calcium–dye complexes becomes slower than the rate of dissociation. For the low-affinity dye, dissociation occurs rapidly, and thus the rising phase of the signal is shorter. Conversely, the slower speed of dissociation of the high-affinity dye leads to a longer rising phase.

Our mathematical model shows that the kinetics of Ca^2+^ transients observed in the presence of dyes with different calcium affinity and different calcium–dye complex dissociation rates could be described using a fixed-buffer concentration of 8.0 ± 0.4 mM and a mobile-buffer concentration of 250 ± 13 μM. Similar results (7.5 mM for slow buffers) have been reported by Bennett et al. (2007), who compared experimental and model-derived presynaptic fluorescence signals during rhythmic stimulation of toad motor nerves, considering the mitochondria and endoplasmic reticulum as fixed buffers. Suzuki et al. (2000) have assumed the existence in the frog nerve terminal cytoplasm of a fixed low-affinity Ca^2+^ buffer at 10 mM concentration. Such fixed buffers can determine the early decay of Ca^2+^ transients after an action potential in crayfish neuromuscular terminals (Lin et al., 2005). Parameters determined from our modeling thus coincide with the values proposed by other authors (Aharon et al., 1994; Suzuki et al., 2000; Bennett et al., 2007).

The idea that the inclusion of fluorescent dyes itself increases the local Ca^2+^ buffering, thus altering the Ca^2+^ dynamics, is often encountered in the literature (e.g., Sabatini et al., 2002). But in our model the interaction of the dye with calcium and diffusion of the dye and the dye-calcium complex in the terminal are simulated separately (Equation 2). Thus, our estimates of buffers concentration are made already taking into account the dye influence on the calcium diffusion. In principle it was shown experimentally (Wu and Betz, 1996) that the calcium dye loading haven't any appreciable influence on the calcium dependent quantal release (parameters of the postsynaptic response or of the frequency of the miniature end-plate potentials do not change). It suggests that endogenous calcium buffers have the buffering capacity much stronger than fluorescent dyes. But our model allows even to check the changes in intracellular calcium distribution at fluorescent dye loading.

The presence of mobile and fixed buffers in the axoplasm explains the time discrepancy between the fluorescent signal and the postsynaptic response (Figures 2, 5). Estimation of the inward calcium current shows that the best agreement between model and experimental fluorescence transients was achieved at a current amplitude of 1.6 ± 0.08 pA and a half-width of 1.2 ± 0.06 ms (Figures 3, 4). Assuming that the conductance of presynaptic N-type calcium channels (which are dominant in frog nerve terminals) is 0.33 pA (Weber et al., 2010), we may conclude that about five channels are activated by a single action potential. These data correspond to the results of Shahrezaei et al. (2006), who showed that from two to six channels open per active zone per action potential; and that fusion of a single vesicle is triggered by the opening of one or two channels.

In summary, the analysis of fluorescence transients in the nerve terminal of the frog neuromuscular junction and the comparison of the kinetics of experimental and model responses allowed us to estimate the intracellular concentrations of fixed and mobile endogenous buffers, the properties of the calcium current, and the kinetics of the changes in calcium concentrations in the nerve terminal.

The high concentration of intracellular calcium buffers, which slow diffusion by binding calcium to cytoplasmic elements, has important consequences for any intracellular Ca^2+^ -dependent process. Diffusion cannot be ignored if the time course of the process studied is on the order of milliseconds, and may contribute to delayed asynchronous release of neurotransmitters (Rahamimoff and Yaari, 1973), calcium-induced calcium release from intracellular calcium stores (Akita and Kuba, 2008), facilitation and augmentation (Miller, 1991), and modulation of Ca^2+^-dependent deactivation of Ca^2+^ channels (Kreiner and Lee, 2006). High concentrations of endogenous buffers modify transmitter release at synapses (Caillard et al., 2000; Matveev et al., 2004).

In conclusion, the concentration of a calcium–dye complex is proportional to the concentration of Ca^2+^ at each point of the terminal volume. When studying small terminals with fast diffusion and a short time to equilibrium, the concentration of Ca^2+^ can be accurately estimated by the first derivative of the smoothed fluorescent signal (Sabatini and Regehr, 1998). Because of the slow “delayed” diffusion and the resulting longer time before equilibrium in the frog motor nerve terminal, the fluorescence signal does not correspond directly to Ca^2+^ concentration in the terminal, particularly near the active zone. However, mathematical modeling allows determination of Ca^2+^ concentration and mobile and fixed buffers concentrations near the membrane of the nerve terminal where the calcium-sensitive exocytosis proteins are placed.

## Funding

This research has been performed under the Russian Government's Program for Competitive Growth of Kazan Federal University and was also supported by a grant of President of Russian Federation “Leading Scientific School” and a grants from the Russian Foundation for Basic Research (13-04-00886; 14-04-00790; 15-04-02983).

### Conflict of interest statement

The authors declare that the research was conducted in the absence of any commercial or financial relationships that could be construed as a potential conflict of interest.

## References

[B1] Aharon S. Parnas H. Parnas I. (1994). The magnitude and significance of Ca^2+^ domains for release of neurotransmitter. Bull. Math Biol. 56, 1095–1119. 7833845 10.1007/BF02460288

[B2] Ahmed Z. Connor J. (1988). Calcium regulation by and buffer capacity of molluscan neurons during calcium transients. Cell Calcium 9, 57–69. 10.1016/0143-4160(88)90025-5 3383224

[B3] Akita T. Kuba K. (2008). Ca^2+^-dependent inactivation of Ca^2+^-induced Ca^2+^ release in bullfrog sympathetic neurons. J. Physiol. 586. 3365–3384. 10.1113/jphysiol.2008.153833 18483065 PMC2538815

[B4] Albritton N. L. Meyer T. Stryer L. (1992). Range of messenger action of calcium ion and inositol 1,4,5-triphosphat. Science 258, 1812–1815. 10.1126/science.1465619 1465619

[B5] Anderson A. J. Harvey A. L. Rowan E. G. Strong P. N. (1988). Effects of charybdotoxin, a blocker of Ca^2+^-activated K+ channels, on motor nerve terminals. Br. J. Pharmacol. 95, 1329–1335. 10.1111/j.1476-5381.1988.tb11772.x 2464391 PMC1854283

[B6] Augustine G. J. (1990). Regulation of transmitter release at the squid giant synapse by presynaptic delayed rectifier potassium current. J. Physiol. 431, 343–364. 1983120 10.1113/jphysiol.1990.sp018333 PMC1181777

[B7] Augustine G. J. (2001). How does calcium trigger neurotransmitter release? Curr. Opin. Neurobiol. 11, 320–326. 10.1016/S0959-4388(00)00214-2 11399430

[B8] Augustine G. J. Neher E. (1992). Neuronal Ca^2+^ signaling takes the local route. Curr. Opin. Neurobiol. 2, 302–307. 10.1016/0959-4388(92)90119-6 1643411

[B9] Bauer P. J. (2001). The local Ca concentration profile in the vicinity of a Ca channel. Cell Biochem. Biophys. 35, 49–61. 10.1385/CBB:35:1:49 11898855

[B10] Bélair E. L. Vallée J. Robitaille R. (2005). Long-term *in vivo* modulation of synaptic efficacy at the neuromuscular junction of Rana pipiens frogs. J. Physiol.569, 163–178. 10.1113/jphysiol.2005.094805 16166159 PMC1464201

[B11] Bennett M. R. Farnell L. Gibson W. G. Dickens P. (2007). Mechanisms of calcium sequestration during facilitation at active zones of an amphibian neuromuscular junction. J. Theor. Biol. 247, 230–241. 10.1016/j.jtbi.2007.03.022 17462674

[B12] Birks R. Huxley H. E. Katz B. (1960). The fine structure of the neuromuscular junction of the frog. J. Physiol. 150, 134–144. 13800900 10.1113/jphysiol.1960.sp006378 PMC1363152

[B13] Borst J. G. Sakmann B. (1996). Calcium influx and transmitter release in a fast CNS synapse. Nature 383, 431–434. 10.1038/383431a0 8837774

[B14] Borst J. G. Sakmann B. (1998). Calcium current during a single action potential in a large presynaptic terminal of the rat brainstem. J. Physiol. 506, 143–157. 10.1111/j.1469-7793.1998.143bx.x 9481678 PMC2230710

[B15] Brigant J. L. Mallart A. (1982). Presynaptic currents in mouse motor endings. J. Physiol. 333, 619–636. 6304288 10.1113/jphysiol.1982.sp014472 PMC1197267

[B16] Bukharaeva E. A. Samigullin D. V. Nikolsky E. E. Magazanik L. G. (2007). Modulation of the kinetics of evoked quantal release at mouse neuromuscular junctions by calcium and strontium. J. Neurochem. 100, 939–949. 10.1111/j.1471-4159.2006.04282.x 17212698

[B17] Bukharaeva E. A. Samigullin D. V. Nikolsky E. E. Vyskocil F. (2002). Protein kinase A cascade regulates quantal release dispersion at frog muscle endplate. J. Physiol. 538, 837–848. 10.1113/jphysiol.2001.012752 11826168 PMC2290098

[B18] Burnashev N. Rozov A. (2005). Presynaptic Ca^2+^ dynamics, Ca^2+^ buffers and synaptic efficacy. Cell Calcium 37, 489–495. 10.1016/j.ceca.2005.01.003 15820398

[B19] Caillard O. Moreno H. Schwaller B. Llano I. Celio M. R. Marty A. (2000). Role of the calcium-binding protein parvalbumin in short-term synaptic plasticity. Proc. Natl. Acad. Sci. U.S.A. 97, 13372–13377. 10.1073/pnas.230362997 11069288 PMC27231

[B20] Chow R. Klingauf J. Neher E. (1994). Time course of Ca^2+^ concentration triggering exocytosis in neuroendocrine cells. Proc. Natl. Acad. Sci. U.S.A. 91, 12765–12769. 10.1073/pnas.91.26.12765 7809118 PMC45520

[B21] Collin T. Chat M. Lucas M. G. Moreno H. Racay P. Schwaller B.. (2005). Developmental changes in parvalbumin regulate presynaptic Ca^2+^ signaling. J. Neurosci. 25, 96–107. 10.1523/JNEUROSCI.3748-04.2005 15634771 PMC6725212

[B22] David J. Barrett J. N. Barrett E. (1997). Stimulation-induced changes in [Ca^2+^] in lizard motor nerve terminals. J. Physiol. 604, 83–96. 10.1111/j.1469-7793.1997.083bf.x 9350620 PMC1159938

[B23] DiGregorio D. A. Vergara J. L. (1997). Localized detection of action potential-induced presynaptic calcium transients at a Xenopus neuromuscular junction. J. Physiol. 505, 585–592. 10.1111/j.1469-7793.1997.585ba.x 9457637 PMC1160037

[B24] Feher J. J. Fulmer C. S. Fritzsch G. K. (1989). Comparison of the enhanced steady-state diffusion of calcium by calbindin-D9K and calmodulin, possible importance in intestinal calcium absorption. Cell Calcium 10, 189–203. 10.1016/0143-4160(89)90002-X 2776187

[B25] Gabso M. Neher E. Spira M. (1997). Low mobility of the Ca^2+^ buffers in axons of cultured Aplysia neurons. Neuron 18, 473–481. 10.1016/S0896-6273(00)81247-7 9115740

[B26] Gilmanov I. R. Samigullin D. V. Vyskocil F. Nikolsky E. E. Bukharaeva E. A. (2008). Modeling of quantal neurotransmitter release kinetics in the presence of fixed and mobile calcium buffers. J. Comput. Neurosci. 25, 296–307. 10.1007/s10827-008-0079-5 18427967

[B27] Helmchen F. Imoto K. Sakmann B. (1996). Ca^2+^ buffering and action potential-evoked Ca^2+^ signaling in dendrites of pyramidal neurons. Biophys. J. 70, 1069–1081. 10.1016/S0006-3495(96)79653-4 8789126 PMC1225009

[B28] Kits K. S. de Vlieger T. A. Kooi B. W. Mansvelder H. D. (1999). Diffusion barriers limit the effect of mobile calcium buffers on exocytosis of large dense cored vesicles. Biophys. J. 76, 1693–1705. 10.1016/S0006-3495(99)77328-5 10049349 PMC1300145

[B29] Klingauf J. Neher E. (1997). Modeling buffered Ca^2+^ diffusion near the membrane, implications for secretion in neuroendocrine cells. Biophys. J. 72, 674–690. 10.1016/S0006-3495(97)78704-6 9017195 PMC1185593

[B30] Kreiner L. Lee A. (2006). Endogenous and exogenous Ca^2+^ buffers differentially modulate Ca^2+^-dependent inactivation of Cav2.1 Ca^2+^channels. J. Biol. Chem. 281, 4691–4698. 10.1074/jbc.M511971200 16373336

[B31] Lin J. W. Fu Q. Allana T. (2005). Probing the endogenous Ca^2+^ buffers at the presynaptic terminals of the crayfish neuromuscular junction. J. Neurophysiol. 94, 377–386. 10.1152/jn.00617.2004 15985697

[B32] Llinás R. I. Steinberg Z. Walton K. (1976). Presynaptic calcium currents and their relation to synaptic transmission: voltage clamp study in squid giant synapse and theoretical model for the calcium gate. Proc. Natl. Acad. Sci. U.S.A. 73, 2918–2922. 10.1073/pnas.73.8.2918 183215 PMC430802

[B33] Llinás R. Sugimori M. Silver R. B. (1992). Microdomains of high calcium concentration in a presynaptic terminal. Science 256, 677–679. 10.1126/science.1350109 1350109

[B34] Luo F. Dittrich M. Stiles J. R. Meriney S. D. (2011). Single-pixel optical fluctuation analysis of calcium channel function in active zones of motor nerve terminals. J. Neurosci. 31, 11268–11281. 10.1523/JNEUROSCI.1394-11.2011 21813687 PMC3412372

[B35] Maravall M. Mainen Z. F. Sabatini B. L. Svoboda K. (2000). Estimating intracellular calcium concentrations and buffering without wavelength ratioing. Biophys. J. 78, 2655–2667. 10.1016/S0006-3495(00)76809-3 10777761 PMC1300854

[B36] Matveev V. Zucker R. Sherman A. (2004). Facilitation through buffer saturation, constraints on endogenous buffering properties. Biophys. J. 86, 2691–2709. 10.1016/S0006-3495(04)74324-6 15111389 PMC1304141

[B37] Miller R. J. (1991). The control of neuronal Ca^2+^ homeostasis. Prog. Neurobiol. 37, 255–285. 10.1016/0301-0082(91)90028-Y 1947178

[B38] Mintz I. M. Sabatini B. L. Regehr W. G. (1995). Calcium control of transmitter release at a cerebellar synapse. Neuron 15, 675–688. 10.1016/0896-6273(95)90155-8 7546746

[B39] Muller A. Kukley M. Stausberg P. Beck H. Muller W. Dietrich D. (2005). Endogenous Ca^2+^ buffer concentration and Ca^2+^ microdomains in hippocampal neurons. J. Neurosci. 25, 558–565. 10.1523/JNEUROSCI.3799-04.2005 15659591 PMC6725329

[B40] Muller M. Felmy F. Schwaller B. Schneggenburger R. (2007). Parvalbumin is a mobile presynaptic Ca^2+^ buffer in the Calyx of Held that accelerates the decay of Ca^2+^ and short-term facilitation. J. Neurosci. 27, 2261–2271. 10.1523/JNEUROSCI.5582-06.2007 17329423 PMC6673482

[B41] Neher E. (1995). The use of fura-2 for estimating Ca buffers and Ca fluxes. Neuropharmacology 34, 1423–1442. 10.1016/0028-3908(95)00144-U 8606791

[B42] Nowycky M. C. Pinter M. J. (1993). Time courses of calcium and calcium-bound buffers following calcium influx in a model cell. Biophys. J. 64, 77–91. 10.1016/S0006-3495(93)81342-0 8431551 PMC1262304

[B43] Pang Z. P. Südhof T. C. (2010). Cell biology of Ca^2+^-triggered exocytosis. Curr. Opin. Cell Biol. 22, 496–505. 10.1016/j.ceb.2010.05.001 20561775 PMC2963628

[B44] Pawson P. A. Grinnell A. D. Wolowske B. (1998). Quantitative freeze-fracture analysis of the frog neuromuscular junction synapse I. Naturally occurring variability in active zone structure. J. Neurocytol. 27, 361–377. 10.1023/A:1006942909544 9923981

[B45] Peng Y. Y. Zucker R. S. (1993). Release of LHRH is linearly related to the time integral of presynaptic Ca^2+^ elevation above a threshold level in bullfrog sympathetic ganglia. Neuron 10, 465–473. 10.1016/0896-6273(93)90334-N 8461136

[B46] Rahamimoff R. Yaari Y. (1973). Delayed release of transmitter at the frog neuromuscular junction. J. Physiol. 228, 241–257. 4346703 10.1113/jphysiol.1973.sp010084 PMC1331238

[B47] Regehr W. G. Atluri P. P. (1995). Calcium transients in cerebellar granule cell presynaptic terminals. Biophys. J. 68, 2156–2170. 10.1016/S0006-3495(95)80398-X 7612860 PMC1282121

[B48] Sabatini B. L. Oertner T. G. Svoboda K. (2002). The life cycle of Ca^2+^ ions in dendritic spines. Neuron 33, 439–452. 10.1016/S0896-6273(02)00573-1 11832230

[B49] Sabatini B. L. Regehr W. G. (1998). Optical measurement of presynaptic calcium currents. Biophys. J. 74, 1549–1563. 10.1016/S0006-3495(98)77867-1 9512051 PMC1299501

[B50] Saftenku E. E. (2009). Computational study of non-homogeneous distribution of Ca^2+^ handling systems in cerebellar granule cells. J. Theor. Biol. 257, 228–244. 10.1016/j.jtbi.2008.12.002 19121636

[B51] Sala F. Hernandez-Cruz A. (1990). Calcium diffusion modeling in a spherical neuron. Relevance of buffering properties. Biophys. J. 57, 313–324. 10.1016/S0006-3495(90)82533-9 2317553 PMC1280672

[B52] Samigullin D. V. Bukharaeva E. A. Vyskočil F. Nikolsky E. E. (2005). Calcium dependence of uni-quantal release latencies and quantal content at mouse neuromuscular junction. Physiol. Res. 54, 129–132. 15717851 10.33549/physiolres.930712

[B53] Samigullin D. V. Vasin A. L. Bukharaeva E. A. Nikolsky E. E. (2010). Characteristics of calcium transient in different parts of frog nerve terminal in response to nerve impulse. Dokl. Biol. Sci. 431, 83–85. 10.1134/S0012496610020043 20506840

[B54] Schmidt H. Brown E. B. Schwaller B. Eilers J. (2003). Diffusional mobility of parvalbumin in spiny dendrites of cerebellar Purkinje neurons quantified by fluorescence recovery after photobleaching. Biophys. J. 84, 2599–2608. 10.1016/S0006-3495(03)75065-6 12668468 PMC1302826

[B55] Schneggenburger R. Neher E. (2005). Presynaptic calcium and control of vesicle fusion. Curr. Opin. Neurobiol. 15, 266–274. 10.1016/j.conb.2005.05.006 15919191

[B56] Schwaller B. (2010). Cytosolic Ca^2+^ buffers. Cold Spring Harb. Perspect. Biol. 2:a004051. 10.1101/cshperspect.a004051 20943758 PMC2964180

[B57] Shahrezaei V. Cao A. Delaney K. R. (2006). Ca^2+^from one or two channels controls fusion of a single vesicle at the frog neuromuscular junction. J Neurosci. 26, 13240–132499. 10.1523/JNEUROSCI.1418-06.2006 17182774 PMC6675009

[B58] Smith G. D. (1996). Analytical steady-state solution to the rapid buffering approximation near an open Ca^2+^ channel. Biophys. J. 71, 3064–3072. 10.1016/S0006-3495(96)79500-0 8968577 PMC1233795

[B59] Stanley E. F. (1991). Single calcium channels on a cholinergic presynaptic nerve terminal. Neuron 7, 585–591. 10.1016/0896-6273(91)90371-6 1657055

[B60] Suzuki S. Osanai M. Murase M. Suzuki N. Ito K. Shirasaki T. K.. (2000). Ca^2+^ dynamics at the frog motor nerve terminal. Pflug. Arch. Eur. J. Phisiol. 440, 351–365. 10.1007/s004240000278 10954322

[B61] Tsentsevitsky A. N. Samigullin D. V. Nurullin L. F. Khaziev E. F. Nikolsky E. E. Bukharaeva E. A. (2014). Presynaptic voltage-dependent calcium channels at the frog neuromuscular junction, in Frogs: Genetic diversity, Neural Development and Ecological Implication, ed Lambert H. (New York, NY: Nova Science Publishers), 179–195.

[B62] Vyshedskiy A. Lin J. W. (2000). Presynaptic Ca^2+^ influx at the inhibitor of the crayfish neuromuscular junction: a photometric study at a high time resolution. J. Neurophysiol. 83, 552–562. 10634895 10.1152/jn.2000.83.1.552

[B63] Wachman E. S. Poage R. E. Stiles J. R. Farkas D. L. Meriney S. D. (2004). Spatial distribution of calcium entry evoked by single action potentials within the presynaptic active zone. J. Neurosci. 24, 2877–2885. 10.1523/JNEUROSCI.1660-03.2004 15044526 PMC6729837

[B64] Weber A. Wong F. Tufford A. Schlichter L. Matveev V. Stanley E. (2010). N-type Ca^2+^ channels carry the largest current: implications for nanodomains and transmitter release. Nature 13, 1348–1350. 10.1038/nn.2657 20953196

[B65] Wu L. G. Betz W. J. (1996). Nerve activity but not intracellular calcium determines the time course of endocytosis at the frog neuromuscular junction. Neuron 17, 769–779. 10.1016/S0896-6273(00)80208-1 8893033

[B66] Wu L. G. Saggau P. (1994). Presynaptic calcium is increased during normal synaptic transmission and paired-pulse facilitation, but not in long-term potentiation in area CA1 of hippocampus. J. Neurosci. 14, 645–654. 7905515 10.1523/JNEUROSCI.14-02-00645.1994 PMC6576817

[B67] Xu T. Naraghi M. Kang H. Neher E. (1997). Kinetic studies of Ca^2+^ binding and Ca^2+^ clearance in the cytosol of adrenal chromaffin cells. Biophys. J. 73, 532–545. 10.1016/S0006-3495(97)78091-3 9199815 PMC1180952

[B68] Yazejian B. DiGregorio D. A. Vergara J. L. Poage R. E. Meriney S. D. Grinnell A. D. (1997). Direct measurements of presynaptic calcium and calcium-activated potassium currents regulating neurotransmitter release at cultured Xenopus nerve-muscle synapses. J. Neurosci. 17, 2990–3001. 9096135 10.1523/JNEUROSCI.17-09-02990.1997 PMC6573664

[B69] Yoshikami D. Bagabaldo Z. Olivera B. M. (1989). The inhibitory effects of omega-conotoxins on Ca channels and synapses. Ann. NY Acad. Sci. 560, 230–248. 10.1111/j.1749-6632.1989.tb24100.x 2545135

[B70] Zhao M. Hollingworth S. Baylor S. M. (1996). Properties of tri- and tetracarboxylate Ca^2+^ dyes in frog skeletal muscle fibers. Biophys. J. 70, 896–916. 10.1016/S0006-3495(96)79633-9 8789107 PMC1224990

[B71] Zhou Z. Neher E. (1993). Mobile and immobile calcium buffers in bovine adrenal chromaffin cells. J. Physiol. 469, 245–273. 8271200 10.1113/jphysiol.1993.sp019813 PMC1143870

