# Supplementary material for: Estimation of presynaptic calcium currents and endogenous calcium buffers at the frog neuromuscular junction with two different calcium fluorescent dyes
Source: Front Synaptic Neurosci. 2015 Jan 7;6:29. doi: 10.3389/fnsyn.2014.00029 (PMC4285738; doi:10.3389/fnsyn.2014.00029)
Supplement: Supplementary file 1 [file Presentation1.PDF]

Supplementary Material

Estimation of presynaptic calcium currents and endogenous calcium buffers at the frog neuromuscular junction with two different calcium fluorescent dyes

Dmitry Samigullin,[1,2,3*] Nijaz Fatikhov,[1] Eduard Khaziev,[1,2] Andrey Skorinkin,[1,4] Eugeny Nikolsky,[1,2,5] and Ellya Bukharaeva[1,2]

[1]Laboratory of the Biophysics of Synaptic Processes, Kazan Institute of Biochemistry and Biophysics, Kazan Scientific Centre, Russian Academy of Sciences, Kazan, Russia
[2]Open Laboratory of Neuropharmacology, Kazan Federal University, Kazan, Russia
[3] Department of Radiophotonics and Microwave Technologies, Kazan National Research Technical University named after A. N. Tupolev, Kazan, Russia
[4]Department of Neurobiology and Department of Radioelectronics, Kazan Federal University, Kazan, Russia
[5]Department of Medical and Biological Physics, Kazan State Medical University, Kazan, Russia

[*]Correspondence:

Dmitry V. Samigullin, Laboratory of the Biophysics of Synaptic Processes, Kazan Institute of Biochemistry and Biophysics, Russian Academy of Sciences, P.O. box 30, Kazan 420111, Russia

Fax: +7(843)2927347

Tel: +7(843)2927647

E-mail: samid75@mail.ru

Running title: Estimation of presynaptic calcium

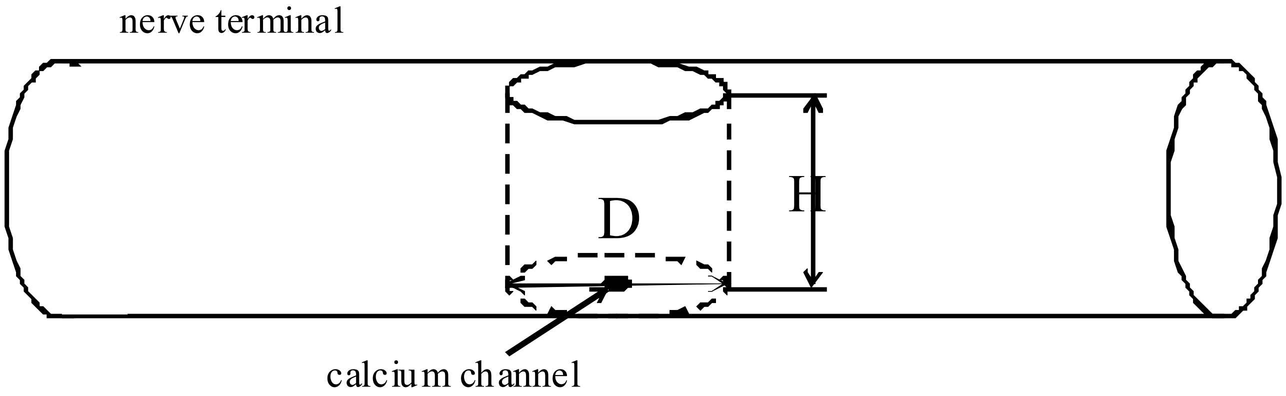

Supplementary Figure 1 (Fig. S1). Schematic representation of the model components. Terminal was represented as a long structure, active zones were modeled as cylinders directed across the terminal. D – diameter of the cylinder, H – its height. Calcium channel was represented as a point source in the center of cylinder's base. We neglected all interactions of channels and considered only one channel in the model of one active zone. We suggested that the inward calcium current in terminal is the linear sum of currents through separate channels.

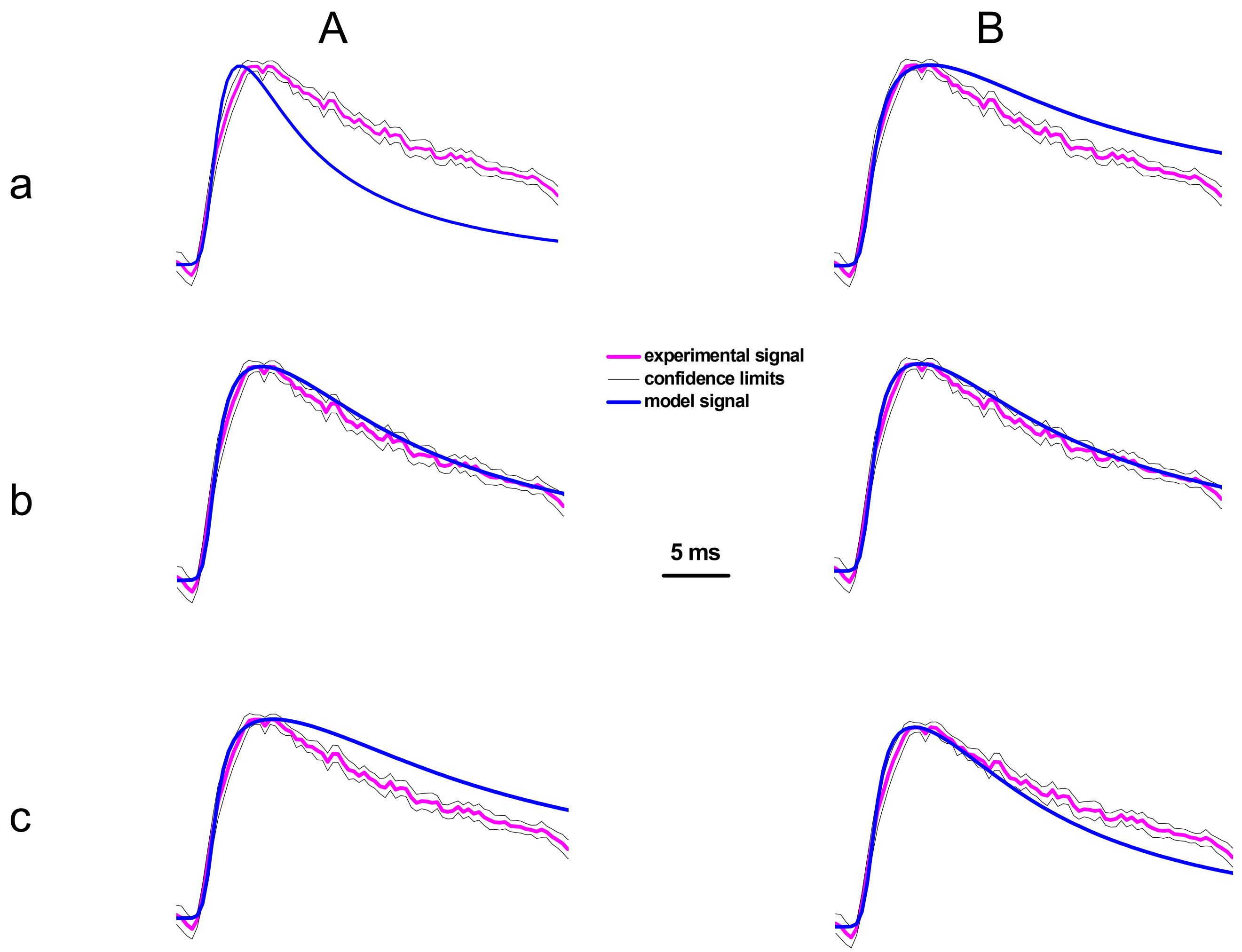

Supplementary Figure 2 (Fig. S2). The selection of fixed and mobile buffer concentrations that provide the best agreement between the model and experimental curves. Superposition of experimental (magenta line) and model (blue line) calcium transients in the terminal loaded with Magnesium Green (experimental traces from Fig. 3) is shown. (**A**) different concentrations of fixed buffer: (**a**) 2 mM (**b**) 8 mM (**c**) 14 mM, concentration of mobile buffer is always 250 µM. (**B**) different concentrations of mobile buffer: (**a**) 50 µM (**b)** 250 µM (**c**) 500 µM, concentration of fixed buffer is always 8 mM. The best approximation of the experimental calcium transients was obtained when the concentrations of fixed and mobile calcium buffers were 8 ± 0.4 mM and 250 ± 13 µM respectively.
